# Perceptions of the preparedness of medical graduates for internship responsibilities in district hospitals in Kenya: a qualitative study

**DOI:** 10.1186/s12909-015-0463-6

**Published:** 2015-10-21

**Authors:** Patricia N. Muthaura, Tashmin Khamis, Mushtaq Ahmed, Syeda Ra’ana Hussain

**Affiliations:** 1 The Aga Khan University, P.O. Box 30270, GPO 00100 Nairobi, Kenya; 2 The Aga Khan University, P.O. Box 38129, Ufukoni Road, Dar es Salaam, Tanzania; 3 The Aga Khan University, P.O Box 258, 00621 Nairobi, Kenya

**Keywords:** Preparedness, Medical school, Internship, District settings, Kenya

## Abstract

**Background:**

Aga Khan University is developing its undergraduate medical education curriculum for East Africa. In Kenya, a 1 year internship is mandatory for medical graduates’ registration as practitioners. The majority of approved internship training sites are at district hospitals. The purposes of this study were to determine: (1) whether recent Kenyan medical graduates are prepared for their roles as interns in district hospitals upon graduation from medical school; (2) what working and training conditions and social support interns are likely to face in district hospital; and (3) what aspects of the undergraduate curriculum need to be addressed to overcome perceived deficiencies in interns’ competencies.

**Methods:**

Focus group discussions and semi-structured interviews were conducted with current interns and clinical supervisors in seven district hospitals in Kenya. Perceptions of both interns and supervisors regarding interns’ responsibilities and skills, working conditions at district hospitals, and improvements required in medical education were obtained.

**Results:**

Findings included agreement across informants on deficiencies in interns’ practical skills and experience of managing clinical challenges. Supervisors were generally critical regarding interns’ competencies, whereas interns were more specific about their weaknesses. Supervisor expectations were higher in relation to surgical procedures than those of interns. There was agreement on the limited learning, clinical facilities and social support available at district hospitals including, according to interns, inadequate supervision. Supervisors felt they provided adequate supervision and that interns lacked the ability to initiate communication with them. Both groups indicated transition challenges from medical school to medical practice attributable to inadequate practical experience. They indicated the need for more direct patient care responsibilities and clinical experience at a district hospital during undergraduate training.

**Conclusion:**

Perception of medical graduates’ unpreparedness seemed to stem from a failure to implement the apprenticeship model of learning in medical school and lack of prior exposure to district hospitals. These findings will inform curriculum development to meet stakeholder requirements, improve the quality of graduates, and increase satisfaction with transition to practice.

## Background

Aga Khan University (AKU) Medical College in East Africa is currently developing an under-graduate medical education (UGME) curriculum for its medical college that is intended to start in Nairobi. The curriculum is outcomes-based, i.e., learning outcomes and competencies are committed to meet the roles of 21^st^ century physicians in East Africa. As part of this program, medical graduates will undertake a mandatory supervised internship for 1 year in an approved hospital. During this time, interns are expected to develop their competencies further as outlined in the Guideline for Interns in Medicine and Dentistry by the Kenya Medical Practitioners and Dentists Board (MPDB) [1].

The MPDB mandates that every doctor shall be required to undergo an internship training program for a period of one (1) year to be conducted on a 3-month rotational basis in each of four main specialties: medicine, surgery, paediatrics/child health, and obstetrics-gynaecology. Successful completion of internship is a requirement for registration and license to practice medicine in Kenya [2].

At the time of this study, only Nairobi University and Moi University were graduating medical students in Kenya. Over the past few years, several new medical colleges have emerged raising the number to nine in 2014. At the time of the present study, approximately three hundred and sixty (360) doctors were graduating every year from Kenyan medical colleges [3]. This number is likely to increase in the near future as new medical colleges graduate their first classes. Over the past 10 years, the number of internship sites has correspondingly increased from 6 to 54 [1]. Of these 29 are at district hospitals. Therefore, the likelihood of a medical graduate training as an intern at a district hospital has significantly increased, whereas most of the clinical experience gained as a medical student is in a teaching hospital which is usually a tertiary referral centre. Yet, little is known about the impact of the shift in practice site.

Several studies have pointed to a gap between medical education or preparation for practice and the actual requirements of medical practice as an intern: many have concentrated on interns’ self-assessment of their competencies [4–7]; some have included the views of supervisors, clinical teams and patients [8–11]. There have been comparisons of different medical schools [12], of graduate students with non-graduate students [13], and of different curriculum designs on preparation for practice [14–16]. Yet other studies have evaluated the contribution of a period of pre-internship training towards preparation for practice [17]. Some work has also gone into junior doctors’ perception of the internship training site [18]. The methodologies used have been questionnaire surveys, interviews and focus group discussions. There are however, limitations on direct extrapolation of the findings from these studies to the East African context where distinctive resource and organizational issues, face medical education and the health service.

There are substantive resource limitation issues in medical schools in Kenya. A parallel stream of medical students who pay tuition fees to supplement funds available to public universities places undue demands on medical education resources [19]. District hospitals in Kenya generally have been described as having limited laboratory and radiology facilities, inconsistent availability of equipment and supplies, a shortage of staff and a lack of information systems [20]. This is in contrast to the relatively well endowed tertiary teaching hospitals. Medical interns may be the only “in house” doctor on duty in a district hospital. The overwhelming responsibility of an intern is in contrast to a predominant observer status as a medical student. Despite their novice status, interns’ duties may extend to cover administrative roles of running the hospital as well as fulfilling clinical responsibilities with a high degree of autonomy and minimum supervision [21]. Finally the context is different from a tertiary teaching hospital in terms of the diseases encountered and patients' socioeconomic and cultural backgrounds.

Keeping these issues in mind, we wanted to assess the perceived magnitude of the gap between medical education and the requirements of practice as an intern in a district hospital in Kenya. Perceptions were elicited from interns and separately from their supervisors about interns’ roles; their competencies; and the shortcomings of the internship sites. Their advice was sought about what medical education should do to overcome deficiencies in medical graduates’ competencies as interns. It is envisioned that the findings and conclusions from this study will inform regional education and health service policies.

## Methods

This study used a qualitative exploratory approach in order to gain insights into the concerns and expectations of interns and supervisors involved in district hospital internship placements. Focus group discussions and semi-structured interviews were conducted with interns and their clinical supervisors to elicit their perceptions on intern preparation in meeting role expectations. Seven district hospitals were sampled constituting 24 % of the 29 district hospitals approved for internship and 13 % of the total (54) internship sites. In order to achieve adequate geographical representation, the aim was to randomly select one district hospital providing internship experience to at least 4 interns from each of the former eight provinces of Kenya. One province did not offer such an experience and was therefore excluded from this study. Two of the authors personally visited each of the seven centres to conduct and scribe the focus group discussions (FGD)/ semi structured interviews. The questions for the FGD were based on a pilot study at the Aga Khan University Hospital in Nairobi with residents who had done their internship 2–3 years ago in various types of hospitals [21]. The questions were modified for the current study which was different in two main respects: (1) the present study was conducted on site and involved interns who were immersed in their internship; (2) a second schedule of questions for intern supervisors was designed to include their views for data triangulation.

The study was approved by the Aga Khan University’s Ethics Review Board. The Director Medical Services in the Ministry of Health was informed. Funding in the form of a grant (URC 12201884) was obtained on the basis of an open competition from the Aga Khan University Research Council. Participation was voluntary. All participants were assured of confidentiality and signed consents were obtained before their participation in the study. Catered lunches were provided for participants as FGDs and semi structured interviews were held over the lunch hour to minimize disruption of clinical activities.

FGD Inclusion criteria: a group of at 4–8 current interns available for 1 h at a MPDB approved district hospital internship training centre; a group of 4–8 consultants supervising current interns at a MPDB approved district hospital internship training centre available for 1 h. FGD Exclusion criteria: Less than 4 current interns or internship supervisors available. Semi structured interviews with intern supervisors were conducted at sites where the minimum number needed for FGD inclusion criteria were not met.

FGDs were held with groups of four or more interns at all 7 sites visited. Separate FGDs were held with supervisors at 2 sites where more than 4 consultants were available. At the other 5 sites where less than 4 consultants were available for FGD, semi structured interviews were held with individual consultants. FGDs were conducted in seminar rooms; semi structured interviews in offices. All responses were transcribed as participants declined recording. Transcriptions and consent forms were stored under lock and key.

Through the FGD/ semi structured interviews, the participants were guided through 4 major aspects of the intern-supervisor experience in the district hospitals. First they explored the roles of an intern at a district hospital. Second, the FGD questions probed for perceptions on how well prepared graduates were for their roles as interns in terms of their general competencies, abilities to perform certain procedures, conduct certain tasks, and manage certain types of clinical problems. Third, a consideration of the challenges of the district hospital site as an internship site was undertaken. Finally, participants’ views were elicited about what medical education should do to overcome any perceived deficiencies in preparing medical graduates for internship in district hospitals. Interns and their supervisors FGDs were conducted separately to allow for data source triangulation. Table 1 shows how comparable questions were posed to interns and their supervisors. In addition, availability of support services was elicited by means of an observation schedule provided to interns for completion. Items on the observation schedule were inferred from an Overview of the Health System in Kenya [22] which states that district hospitals should provide: “curative and preventive care and promotion of health, quality clinical care by a skilled and competent staff, treatment techniques such as surgery, laboratory and other diagnostic techniques, inpatient care, training and technical supervision to health centres, 24 h services in obstetrics and gynaecology, child health, medicine and surgery, including anaesthesia, and accident and emergency services”.

**Table 1:** Comparable questions posed to interns and their supervisors

Questions to interns	Questions to interns’ supervisors
● Do you feel the following are the main functions of an intern in a Kenyan context?	● Do you feel the following are the main functions of an intern in a Kenyan context?
○ Diagnose and manage common clinical problems	○ Diagnose and manage common clinical problems
○ Request investigations and interpret results of common tests	○ Request investigations and interpret results of common tests
○ Prescribe medicines	○ Prescribe medicines
○ Perform common procedures	○ Perform common procedures
○ Recognize and treat common emergencies	○ Recognize and treat common emergencies
○ Resuscitate unstable patients	○ Resuscitate unstable patients
○ Monitor patient’s clinical progress	○ Monitor patient’s clinical progress
○ Communicate with patient/family	○ Communicate with patient/family
○ Obtain informed consent	○ Obtain informed consent
○ Write discharge summary and coordinate patient follow up	○ Write discharge summary and coordinate patient follow up
○ Balance work priorities	○ Balance work priorities
○ Communicate with the clinical team	○ Communicate with the clinical team
○ Seek advice/ know when to refer others?	○ Seek advice/ know when to refer others?
● How well prepared were you for these roles?	● How well prepared were the interns for these roles?
● What *procedures* were you expected to do and how well prepared were you to do them?	● What *procedures* would you expect an intern to do and how well prepared were they to do them?
● How skilled were you to perform the following *tasks*:	● How skilled were interns to perform the following *tasks*:
○ Diagnose and manage common clinical problems	○ Diagnose and manage common clinical problems
○ Request investigations and interpret results of common tests	○ Request investigations and interpret results of common tests
○ Prescribe medicines	○ Prescribe medicines
○ Perform common procedures	○ Perform common procedures
○ Recognize and treat common emergencies	○ Recognize and treat common emergencies
○ Resuscitate unstable patients	○ Resuscitate unstable patients
○ Monitor patient’s clinical progress	○ Monitor patient’s clinical progress
○ Communicate with patient/family	○ Communicate with patient/family
○ Obtain informed consent	○ Obtain informed consent
○ Write discharge summary and coordinate patient follow up	○ Write discharge summary and coordinate patient follow up
○ Balance work priorities	○ Balance work priorities
○ Communicate with the clinical team	○ Communicate with the clinical team
○ Seek advice/ know when to refer	○ Seek advice/ know when to refer
● Which groups of *common clinical problems* did you feel competent to deal with or not?	● Which groups of *common clinical problems* do you feel interns are competent to deal with or not?
○ Maternal problems related to pregnancy and child birth	○ Maternal problems related to pregnancy and child birth
○ Neonatal problems	○ Neonatal problems
○ Problems of early childhood	○ Problems of early childhood
○ Unstable clinical problems in adults requiring hospitalization	○ Unstable clinical problems in adults requiring hospitalization
○ Acute illness requiring intensive care	○ Acute illness requiring intensive care
○ Stable clinical problems including chronic illnesses that could be managed on an ambulatory basis	○ Stable clinical problems including chronic illnesses that could be managed on an ambulatory basis
○ Medical, surgical, obstetrical and paediatric emergencies	○ Medical, surgical, obstetrical and paediatric emergencies
● What were your main challenges as an intern?	● What do you feel are the main challenges facing an intern at a district hospital??
● Was there anything you felt ill prepared for?	● In our discussion with interns they expressed the following about their experience of medical school; what do you think?
	● Is there anything else you would like to say about your experience with interns that we have not covered?
● How well are you supported as an intern?	● What support does an intern at your hospital receive?
● Are you planning to pursue PGME?	
● If you were planning UGME curriculum, what would you include to ensure interns are effective in district hospitals in Kenya?	● If you were planning UGME curriculum, what would you include to ensure interns are effective in district hospitals in Kenya?

The semi-structured interview and FGD schedules were analyzed using aspects of grounded theory described by Strauss and Corbin [23]. Through systematic reading and rereading of the results, emerging common themes were emphasized. In the analysis, patterns were identified and trends highlighted across the various data sets obtained from interns, supervisors, and the observation schedules related to the district hospitals, in order to triangulate the information. A comparison was made of interns’ and clinical supervisors’ perceptions of interns’ abilities and the support accorded to them as well as their views on medical education.

## Results

There were a total number of 32 interns who participated in focus groups at the different sites. There were a total number of 18 supervisors who participated. The supervisors were consultants at the district hospitals, who were registered as specialists by the MPDB in each discipline required for internship namely General surgery (3), Internal medicine (6), Paediatrics (3), Obstetrics and Gynaecology (6). Interns had graduated from medical colleges in Kenya (University of Nairobi and Moi University), Uganda and medical colleges in Russia, Ukraine and China.

This section further analyses responses to the 4 major areas related to interns’ and their supervisors’ perceptions regarding: (1) interns roles; (2) competencies in relation to these roles; (3) service, training and social support provided at district hospitals, and (4) advice to medical schools to address perceived deficiencies in competence.

### Interns’ roles

Interns and their clinical supervisors agreed with the roles proposed for interns: assessing and investigating patients, prescribing, performing procedures, managing emergencies, monitoring patients’ progress, communicating with patients and staff, obtaining informed consents, writing discharge summaries, and balancing work priorities (see Table 1). The interns added two other functions: presenting cases during ward rounds and supervising ‘clinical officer students and interns’ (Clinical officers are non-physician clinicians or mid-level health care providers who receive less training than physicians, have a more restricted scope of practice and are accredited by the Clinical Officers Council of Kenya). The supervisors also added two functions: attending outpatient clinics and performing surgical procedures e.g., caesarean section.

### Perceived defects in competencies

The interns’ and their supervisors’ perceptions related to interns’ general skills, abilities to perform tasks, and their management of clinical problems in different settings were compared.

#### Clinical assessment skills

The interns felt confident about their history taking and physical examination skills. The supervisors however felt that interns needed to improve their physical examination skills particularly pelvic examination. They also felt interns needed to improve their clinical reasoning skills for diagnosis.

#### Investigation skills

While interns were confident about requesting appropriate investigations and interpreting test results, their supervisors felt that interns ordered too many investigations reflecting an approach acquired during clerkship (in teaching hospitals) where undue reliance was placed on tests for making a diagnosis rather than on a good history and physical examination. The supervisors also perceived deficiencies in interns’ interpretation of test results.

#### Procedure skills

Interns described filling log books during their clerkships as evidence of performing ward procedures. They did not describe undergoing objective assessment of their procedure skills. Both interns, albeit to a lesser extent, and their supervisors felt there were deficiencies in performing routine ward procedures such as drawing blood; placing intravenous lines, nasogastric tubes and urinary catheters; and doing lumbar punctures and pleural and ascetic taps.

Supervisors felt that during the internship, interns needed to learn to do surgical procedures independently such as wound debridement, caesarean section, hernia repair, lymph node biopsy and laparotomy for ruptured ectopic pregnancy. However, they felt that interns were ill prepared even for basic surgical skills such as skin suturing.

#### Prescribing skills

Interns readily acknowledged their deficiency in prescribing skills, which was affirmed by their supervisors – “(we) don’t expect fresh graduates to know doses, but they should know drugs for common problems”. The supervisors also felt that interns had a poor understanding of drug interactions.

#### Basic communication and interpersonal skills

The interns said they were not taught communication skills in medical college. They felt especially deficient in communicating with nurses and felt that the senior nurses had an “attitude”, that interns’ views did not count and their prescribing was often challenged. Interns also felt unprepared to counsel patients and found it difficult to overcome patients’ deficient understanding of their medical illness as a result of a low level of literacy.

The supervisors were scathing about interns communication and interpersonal skills –“no communications skills”; “not trained in interpersonal skills”. The supervisors felt that interns were fearful about communicating which led to issues with patient management.

#### Performance of various tasks

Interns accepted that initially they had difficulty prioritizing tasks. The supervisors felt that “determining the sickest patients (was) a serious challenge” for the interns.

Although the interns attributed their deficiency in monitoring patients’ clinical progress to an excessive workload, their supervisors pointed to specific weaknesses in reading partographs and charting fluid intake and output. Both parties agreed that interns were weak in obtaining informed consents. Likewise, writing discharge summaries was a problem.

#### Managing emergencies

Interns were confident about recognizing emergencies but were not confident about managing them. In particular they felt their resuscitation skills were lacking which was compounded by the lack of availability of suitable resuscitation equipment. The only exceptions were paediatric and neonatal emergencies thanks to the Emergency Triage and Treatment (ETAT) training they had received as medical students. Interns expressed a need for other emergency medicine training courses in cardiac, trauma and obstetrical life support. The supervisors were in full agreement with the interns’ perceptions.

#### Ambulatory care skills

The interns were least prepared to manage chronic illness on an ambulatory basis. They felt that as medical students they were mostly involved with inpatients and had attended outpatient clinics only once a week. The supervisors felt that interns “over admit” initially. Similarly follow up of patients discharged from the ward was often delegated to ‘clinical officer interns’.

### Perceptions of working conditions at district hospitals

#### Clinical support services and equipment

Both interns and their supervisors felt that diagnostic support and the medications available at the district hospital were deficient. They also felt there was a lack of resuscitation equipment and critical care facilities.

However, when interns’ observation about the availability of support was elicited by providing a list of items and asking whether a given item was ‘always available’, ‘usually available’, ‘rarely available’, or ‘not available’, it seemed that x-rays and ultrasound were regarded most often as ‘usually available’ whereas CT scans and radiologists were ‘not available’. Similarly, most of the drugs with the exception of cancer drugs were scored equally as ‘always available’ or ‘usually available’. Ward equipment was scored equally as ‘usually’ or ‘always available’. The operating theatre, anaesthetist, and general surgery and obstetrical instruments sets were scored equally as ‘usually available’ or ‘always available’. Most lab tests were also scored equally as ‘usually’ or ‘always available with the exception of histology.

#### Supervision

Whereas the interns felt that supervision was inadequate, their supervisors felt that the interns did not communicate well and in a timely manner. The supervisors felt they were not only supervising but also teaching on rounds and organizing CME sessions. However, the supervisors acknowledged they could have done a better job of mentoring the interns. Both parties agreed that learning resources in district hospitals were inadequate and that district hospitals were not organized for training purposes.

#### Social support

Both parties agreed that the living accommodation, food and recreation for interns were inadequate. Interns felt they were poorly paid and did not have enough time off. The supervisors said “there is a lot that goes into making sure (that) at the end of the internship there is a competent doctor”. However, they felt that their contributions as supervisors were not recognized and that they were not adequately rewarded.

### Advice for medical schools

#### More practical experience

From both parties there was a plea for providing more practical experience in medical college. Many interns felt that while they had acquired a theoretical knowledge base, they lacked practical experience including training to perform procedures. This sentiment was echoed by the interns’ supervisors who said, “We are teaching them things they should have learned in medical school”.

The quest for more practical experience was reinforced by interns’ statements concerning student class and group size and the need for closer supervision in medical school. The interns pointed out, that medical students needed more direct patient care responsibility and supervision during their clerkships. The supervisors said that interns “should have a better understanding of professional practice”.

Both groups of informants recommended more obstetrical and surgical exposure to learn procedure skills, more experience of managing paediatric patients, experience of managing emergencies and performing resuscitation, and more exposure to outpatient care during medical school.

#### Experience at a district hospital

The recommendation for rotation of students through district hospitals during their senior years was loud and clear from both parties implying that medical students should have experience of the working conditions in district hospitals. The interns felt that the abrupt severance from an academic environment came as a “shock” especially the gruelling hours of work.

#### Skills improvement

The skills proposed for greater emphasis by both interns and supervisors were: physical examination, clinical reasoning, appropriate investigations and interpretation of tests, ward procedure skills, prescribing skills and communication skills. Teamwork skills especially inter-professional collaboration was also emphasized. Some of the supervisors proffered that Moi University medical graduates had better practice skills in comparison to those graduating from more traditional colleges.

#### Selection of students

While interns felt that medical students should be helped to develop coping skills to manage stressful situations such as dying patients, paradoxically, interns’ supervisors wanted selection of tougher students who could cope with life as a doctor.

## Discussion

The present study explored perceived adequacy in the preparation of recent Kenyan medical school graduates for their roles as interns in district hospitals in order to inform the emergent design of the Aga Khan University undergraduate medical education curriculum. As the majority of graduates are likely to be posted to district hospitals, their ability to function effectively in district hospital settings is an important curriculum objective. The conditions of work i.e., clinical support services, training and social support, which interns face in these settings are also important considerations as they influence performance.

### Perceptions of competencies in relation to interns’ roles

#### Clinical skills

Even in developed countries medical graduates have been perceived to be deficient in the basic clinical skills of history taking, physical examination and clinical reasoning, indicating a failure of the medical curriculum [24, 25]. Defective clinical reasoning continues to be an important cause of diagnostic error [26]. Arguably, basic clinical skills and clinical reasoning assume greater importance in district hospitals in Kenya because interns need to be more autonomous. Also they have limited access to diagnostic tests. This places an onus on medical schools in Kenya and other developing countries to ensure that students’ basic clinical skills including clinical reasoning, are adequately developed.

#### Investigations

The cost and availability of tests in the relatively resource poor environment of district hospitals are important considerations for reducing the reliance on investigations. Indeed, education programmes that emphasize appropriate ordering of investigations have been shown to reduce health care costs without negatively impacting patient care [27, 28]. Such programs should be adopted in teaching hospitals in Kenya. The absence of pathologists and radiologists in district hospitals in Kenya place a responsibility on medical schools to ensure that students learn to interpret test results.

#### Procedures

Medical students are expected to learn to perform basic ward procedures. In the current study, interns described filling log books as evidence of achieving competence in medical school but did not describe undergoing objective skills assessment. We propose that validated competency assessment tools requiring Direct Observation of Procedure Skills should supplement log books in both medical schools and during internship training in order to confirm demonstrable competence in performing procedures.

Internship supervisors’ expected interns to do surgical procedures independently after an orientation period. Heavy workloads in district hospitals as compared with the size of the medical staff may explain why interns are obliged to perform surgery independently, but does not justify compromise in the quality of care that might occur unless the interns were carefully supervised. In the present study there was debate about the availability of supervision; the interns claimed that it was not readily forthcoming while their supervisors disagreed. If the high Maternal Mortality Ratios in Africa are anything to go by, then certainly a lack of well-trained/ well-supervised staff in obstetrics is at least part of the reason [29].

#### Prescribing

Many studies have alluded to new medical graduates’ deficiency in prescribing skills in both developed [30, 31] and developing country contexts [32]. Prescribing errors are responsible for a substantial proportion of all medication errors and contribute to injury and death [33].

Many ways of improving prescribing efficiency have been proposed e.g., the use of smartphone pharmaceutical apps to support hospital based prescribing and pharmacology education. However, this approach has to be accepted with caution as the apps designers may not have sufficient medical knowledge [34]. Similarly, a limited ‘student formulary’ to enable learning around a core list of commonly used drugs has been advised for medical students [35]. In a systematic review of educational interventions to improve prescribing by medical students and junior doctors it has been shown that ‘The WHO Good Prescribing Guide’ is effective across a wide range of trainees in international settings [36]. The increasing use of multiple drugs as a result of the rising incidence of NCD related comorbidities necessitates awareness of drug interactions.

#### Basic communication and interpersonal skills

A lack of efficient communication between interns and nurses was perceived in the present study. It has been observed that medical errors are commonly due to communication errors between caregivers [37]. Hence, best practice tools for standardized health care like SBAR (Situation, Background, Assessment and Recommendation) have been introduced to minimize errors in North America and should be considered for adoption in hospitals in East Africa. The difficulties interns faced in communicating with their supervisors might reflect insufficient opportunity to practice such communication during the clerkships. A change in the nature of the clerkship to an apprenticeship model with direct patient care responsibility would require students to communicate with their immediate supervisors.

In a multi-ethnic population with variable levels of education there are substantial challenges to obtaining an informed consent or developing an effective therapeutic relationship with patients. Many international studies have developed and assessed the skills of medical trainees to communicate with colleagues and especially with patients who have low levels of health literacy [38–40]. Contextual studies with socioeconomically and culturally diverse patient populations in Kenya would be required in order to develop effective programs for communication training.

#### Emergencies

Most new interns felt they were well prepared for new-born and paediatric emergencies, as a result of the ETAT (Emergency Triage and Treatment) training they received in medical school. This perception is supported by a study at the Kenyatta National Hospital, which demonstrated significant improvement in documented clinical practices following the introduction of ETAT [41]. The interns in our study expressed the need for other emergency medicine courses such as ATLS (Advanced Trauma Life Support), ACLS (Advanced Cardiovascular Life Support) and ALSO (Advanced Life Support in Obstetric). Dauphin-McKenzie et al. [42] have demonstrated how an ALSO orientation better prepared new residents for managing obstetrical and gynaecological emergencies.

Unfortunately in public hospitals including national referral hospitals in Kenya, the sickest patients are often transferred to the ward without any resuscitation efforts in the emergency room [43]. The chances of this happening could potentially be reduced if medical and other personnel were uniformly trained in resuscitation. Although ACLS and ATLS training and implementation require expensive equipment, basic resuscitation measures for trauma and obstetrics do not.

### The district hospital as a training site

Internship is a time to apply knowledge and skills learned in medical school and to learn new skills under supervision in preparation for postgraduate education in a field of choice. This represents the continuum of medical education. Accordingly, the Kenyan MPDB has laid down training objectives of the internship*.* The suitability of the district hospital as an internship site however, remains contentious.

#### Availability of clinical support services

There seems to be a contradiction between interns’ and their supervisors’ impressions of lack of support services and equipment at the district hospitals and interns’ observations based on an observation schedule or check list that seemed to suggest that most things were either usually or always available. However, the available support services at a district hospital may not meet the needs for adequately diagnosing and managing patients with common clinical problems encountered at that level of service and for referring patients appropriately. The supervisors’ comments based on the observation schedule may have shed more light on availability vs. needs, but unfortunately were not elicited. Thus despite the interns’ opinions based on the observation schedule, the service facilities could still be well short of training expectations.

#### Supervision

Perceptions about the extent of supervision differed widely between the interns and their supervisors. This difference in perception may be attributed to the “hidden curriculum” [44] which embraces an unspoken tradition of praising trainees as being “strong” when they are able to carry heavy workloads with little supervision, and “weak” when they call for help, or even when they realize they need help. Steep hierarchies in medicine, not limited to Kenya, have caused trainees to be reluctant in voicing their concerns in critical situations [45]. Hafferty advises that changes to medical education are inadequate if undertaken only at the level of the curriculum; necessitating a redress of the hidden curriculum both implicitly and explicitly [46]. The district hospital training site should be seen as a learning environment and reform should consider what students learn informally as well as formally with equal valuing.

#### Inter-professional teamwork

Both interns and their supervisors in the present study observed that interns lacked interpersonal skills especially when it came to working with the nursing staff. Inter-professional education has been strongly advocated by The Global Independent Commission on Education of Health Professionals for a new Century as a means of addressing this deficiency [47]. It involves students from two or more professions learning together, especially about each other’s roles, and respectfully interacting with each other on a common educational agenda. Although effective, collaborative work within a cohesive group should start in professional schools, a team approach must actually be practiced in the workplace to set the example. The aim should be to set up effective communities of practice in all practice settings.

#### Continuity of care

From the present study, it appears that the interns are providing episodic care which is largely inpatient based; they are inadequately exposed to outpatient clinics. In this respect it was no different from their clerkship experience. By contrast training is dependent on providing continuities of patient care, supervision and membership of a clinical team [48–50]. This could potentially be achieved during internship given that the one year of internship is equally divided between 4 specialties provided that an outpatient experience is also built in. Thus the concepts of inter-professional collaborative teamwork and of continuity of supervision and patient care are applicable across the continuum of medical education from the clerkships through to internship and residency training.

#### Social support

Of the many challenges to training in district hospitals, we would like to discuss the issue of social support which was brought up by both groups of informants in the present study. The interns described the internship as “a shock to the system” and wished they had been better prepared for the gruelling hours of work. Work probably became even more unbearable as social support in the form of good meals, decent accommodation, and adequate time off work were not forthcoming. The sense of inadequate remuneration added to the discontentment.

To some extent interns’ unhappiness could have been alleviated if internship supervisors had served as mentors. However, this did not happen. Providing career guidance in medical school, including information about the internship and how it serves as a step towards residency training, could perhaps also alleviate the sense of disenfranchisement.

Internship supervisors wished they were recognized for their contributions to interns’ training. They felt that it was only fair that they should receive stipends for teaching. They also wished that internship training sites were better equipped for proper training.

The new Constitution of Kenya has devolved health care management which was previously in the hands of the national government, to the level of the counties with the expectation that this will lead to better equipment, staffing and management of district hospitals. It seems that purchase and installation state of art medical equipment in county hospitals has already started [51]. This may contribute towards greater professional satisfaction of the supervisors and lead to a more conducive learning environment. The Kenyan Medical Practitioners and Dentists Board should also do more to develop stringent criteria for district hospitals to serve as internship training sites and accredit only those sites that strictly meet the requirements. One of the criteria should be the educational training of clinical supervisors.

### Advice for medical schools

Both interns and their supervisors insisted on more practical experience for medical students and clerkship experience in a district hospital setting. Illing et al. [12] have attributed the lack of medical graduates’ preparedness to a failure to implement the apprenticeship model of learning or ‘learning on the job’ during medical education. In this model which is based on situated learning theory, students learn by engaging in the processes of patient care within a well-knit clinical team. Illing et al. point out that in the absence of such engagement the skills learned through real life experience are particularly deficient viz. ‘ward work, being on call, management of acute clinical situations, prescribing, clinical prioritizations, time management and dealing with paper work’.

The Longitudinal Integrated Clerkship which has become popular also gives importance to direct patient care within a clinical team and provides for longitudinal patient follow up [52]. Some medical schools provide a sub-internship which allows students greater responsibility for patient management than in a normal clerkship, while still ensuring supervision [17].

The decentralization of clinical training from tertiary hospitals to district hospitals, which has just started in Kenya [53], will very likely provide students the opportunity for direct patient care responsibility. However, it requires significant investment of faculty time for supervision. At present in Kenya, the large student numbers compared with supervised clinical sites is one of the main reasons for failure to provide medical students supervised patient care responsibility. This contrasts with the West, where concern for patient safety is regarded as a key reason for not involving medical students as responsible team members [12].

Our internship supervisors opined that Moi University medical graduates had better practical skills in comparison to graduates from more traditional colleges. This finding was corroborated in a cross sectional study in Kenya [54] in which medical graduates with a PBL background felt they were better prepared for their roles as interns compared with their peers from traditional curricula.. However, the superiority of PBL in this regard is not a widely accepted [14]. Most modern curricula adopt an outcome-based approach in which learning outcomes and competencies are in keeping with the roles required in a given health care system. Instructional and assessment strategies are then aligned to the acquisition of these competencies. The trouble is that PBL and outcome-based curricula require a strong resource base and rigorous programme management for their success [55], things that are hard to come by in Kenya.

#### Strengths and weaknesses of the present study

The main strength of this study is the representativeness of study sites. Currently district hospitals comprise over 50 % of internship training sites in Kenya. There was also adequate sampling of district hospitals from all the geographic areas with one exception.

Perceptions of both interns and internship supervisors were obtained. There seemed to be general agreement between interns and their supervisors that interns had significant weaknesses in their skills. However, the supervisors were more generally critical of the interns’ competencies whereas the interns were more specific about their weaknesses. Others have also observed discrepancies between interns’ self-assessments and their supervisors’ assessments of them [10]. Furthermore, the supervisors seemed more concerned about interns’ skills’ deficiencies that directly affected their work as consultants e.g., ability to perform surgical procedures; in other words the supervisors’ perceptions were tainted by a service perspective. Similarly the interns were more concerned about things that mattered most to them e.g., their abilities to counsel patients and families, and teach ‘clinical officer interns’. From a training perspective a more holistic evaluation of interns’ competencies will be necessary.

Conducting focus group interviews eliminated problems of poor response/participation as may be encountered with mail or online questionnaires; and the group dynamics provided additional insights.

##### Weaknesses

Interns’ observation of the available clinical support services using an observation schedule provided a reference frame against which to judge perceptions. However, an assessment of the appropriateness and quality of the services which the internship supervisors may have been better qualified to comment on was not elicited.

This study did not investigate the backgrounds of the supervisors in terms of their teaching background and experience. Further studies are needed to elicit the competence of internship supervisors to provide training to medical interns.

The present study did not permit a distinction to be made based on the medical school or type of curriculum. The interns had graduated from different medical schools in Kenya and other countries. Within Kenyan medical schools only Moi University Medical College provides PBL whereas other schools provide more traditional curricula. A national medical licensing examination to compare standards between medical schools does not exist in Kenya and at any rate would not necessarily reflect performance at work.

Interns were interviewed at different points in their internship. Not all interns had the same experience of specialties to which they could relate their skills. A prospective study to elicit perceptions at the start and at the end of internship might provide more uniform information.

Internship supervisors raised concerns over interns’ professionalism. Their comments touched on issues of dress, punctuality, availability for, and response to calls points to the challenges of medical schools to impart good values and fitness to practice as described by the General Medical Council [56]. Professionalism was not a focus of the present study.

#### Further studies

The present study elicited perceptions of preparedness rather than actual competence and similarly perceptions of support rather than objective measures of support. Assessment of performance at the workplace would be ideal to evaluate the preparedness of medical graduates for internship. Leaving aside the difficulty of conducting work-based assessments at various sites, this would necessitate synchronizing events such that all the interns were having similar experiences at the time of assessment, preferably at the beginning of their internship in order to exclude effects of learning during internship.

## Conclusion

Medical graduates’ unpreparedness seemed to stem mainly from a failure to implement the apprenticeship model of learning in medical school and the persistence of this problem into the internship. Although failure to implement a ‘learning on the job’ model may be widespread its adverse impact is more marked in East Africa and its root causes related to resource insufficiency are more intractable. In Kenya, the MPDB which bridges education and service could play an important role in aligning the two, such that medical education prepares graduates for real life responsibility as interns and the district hospitals offer adequate support for interns to fulfil that responsibility. A stimulating internship experience is an important prelude to further residency training. These findings will inform the development of the Aga Khan University’s undergraduate medical education curriculum for East Africa to meet stakeholder requirements, improve quality of graduates, and increase satisfaction with the medical school to practice transition.
